# Phase sensitive inversion recovery with simultaneous dark fat rendering by virtual chemical inversion

**DOI:** 10.1186/1532-429X-14-S1-P274

**Published:** 2012-02-01

**Authors:** Elizabeth Jenista, Wolfgang G Rehwald, Han W Kim, Stephen Darty, Deneen Spatz, Enn-Ling Chen, Raymond J Kim

**Affiliations:** 1 Cardiolgy, Duke University, Durham, NC, USA; 2 Siemens Healthcare Cardiac MR R&D, Chicago, IL, USA

## Background

On delayed-enhancement (DE-CMR), both fat and infarcted myocardium appear bright making them difficult to differentiate. Standard chemical shift fat-suppression (FatSatCS) consists of a fat-frequency selective saturation followed by readout. This approach is suboptimal in clinical DE-CMR sequences which typically have long readout times (100-200ms) and as a result, fat magnetization has significantly recovered (T1 = 290ms, 3T) when the center of k-space is acquired for linear reordering. Centric reordering can overcome this limitation, but at the cost of blurring artifacts.

The phase sensitive inversion recovery (PSIR) variant of DE-CMR avoids the need to precisely set inversion time through the acquisition of a reference data set interleaved between image acquisitions. We hypothesized that modifying the reference acquisition via a Dixon-type approach could provide PSIR images that simultaneously show dark fat without added acquisition time. The result is a virtual chemically selective inversion (FatSatVCSI), without increased SAR and at no cost to signal-to-noise from pulse imperfections.

## Methods

DE-CMR images in phantoms and patients (n=7) were acquired at 3T (Siemens) with a segmented gradient-echo readout. In phantoms, we studied the relationship of readout duration (by varying the number of lines per segment) to fat signal. FatSatCS efficacy was studied for both centric and linear reordering. In patients, three sequential PSIR images were acquired: no fat suppression, FatSatCS and the FatSatVCI with the same parameters including inversion time. Regions of interest (ROIs) were drawn in pericardial and subcutaneous fat and left ventricular blood pool. The performance of each technique was evaluated by comparing the fat-to-blood signal ratios.

## Results

**Figure 1:**
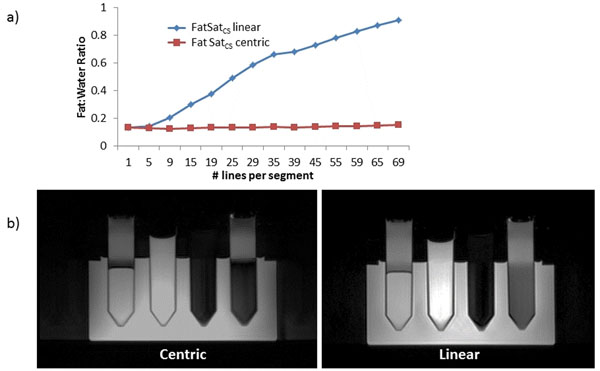
a)Fat suppression efficacy as a function of the number of segments for DE-CMR. Fat:Water ratio is calculated by dividing the fat signal by the TI=210 water signal for an increasing readout times. b) From left to right the tubes are: fat + water (TI=300ms), water (TI=210ms), Water (TI=970ms), and oil . Comparison of centric and linear FatSatCS methods with a clinically relevant readout length (200 ms). Note in the centric that while the fat suppression remains excellent, there is significant blurring.

**Figure 2:**
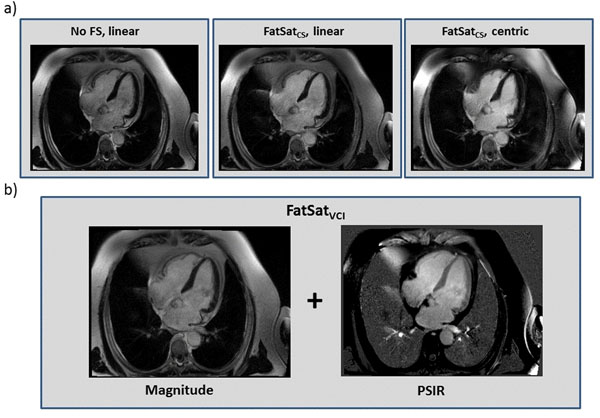
Comparison of the classic chemical shift fat saturation method (both centric and linear reordering) to the proposed method. A) FatSatCS method (centric or linear) shows only a slight reduction in fat signal intensity. B) The new method, FatSatVCI gives both the standard delayed-enhancement image as well as a black fat PSIR image at no additional cost.

Phantom results demonstrate that increasing readout time reduces the efficacy FatSatCS with linear reordering, but not for centric (figure 1a). With centric reordering, significant blurring artifacts occurred (figure 1b) with clinically relevant readout times. In patients, FatSatCS (linear) had no effective fat saturation (1.05±0.08 and 1.07±0.03 for no fat saturation, p = 0.29). FatSatVCI provided effective fat suppression (0.54±0.06, P<0.001 for all comparisons). Figure 2 shows patient images of all fat suppression techniques including linear and centric reordering.

## Conclusions

By modifying the reference acquisition of a PSIR scan, we provide PSIR images with and without fat suppression in a single acquisition. No additional data acquisition, RF pulses, or increased scan time is required.

## Funding

NIH grant 5R01HL064726-07.

